# Management of intra-operative acute pulmonary embolism during general anesthesia: a case report

**DOI:** 10.1186/s12871-017-0360-0

**Published:** 2017-05-26

**Authors:** Yuanyuan Mao, Shuai Wen, Gezi Chen, Wei Zhang, Yanqiu Ai, Jingjing Yuan

**Affiliations:** 1 Department of Anesthesiology, First Affiliated Hospital of Zhengzhou University, No.1 East-JianShe Road, Zhengzhou, 450052 China; 2 Department of Modern Education Technology Center, Henan Medical College, No.8 Shuanghu Road, Longhu, Zhengzhou, 451191 China

**Keywords:** Intra-operation, Acute pulmonary embolism, General anesthesia, Multidisciplinary consultation

## Abstract

**Background:**

Acute pulmonary embolism (APE) can be life-threatening. Early detection is even more difficult for patients under general anesthesia as common symptoms are not available and the pathophysiological course of intra-operative APE is influenced by procedures of surgery and anesthesia, which makes patients under general anesthesia a distinctive group.

**Case presentation:**

We report a case of APE during orthopedic surgery under general anesthesia. A 64-year-old female with atrial fibrillation and surgical history of varicosity underwent total right hip replacement surgery under general anesthesia. No arterial or deep vein thrombosis (DVT) was found prior to the surgery, but APE still occurred intraoperatively. The sudden decrease in P_ET_CO_2_ and increase in PaCO_2_ combined other clues raised the suspect of APE, which is further evidenced by transesophageal echocardiogram (TEE). Multidisciplinary consultation was started immediately. After discussion with the consultation team and communication with patient’s family members, anticoagulation therapy was started and IVC filter was placed to prevent PE recurrence. The patient went through the operation and discharged uneventfully 30 days later.

**Conclusions:**

Pulmonary embolism is a rare and potentially high-risk perioperative situation, with a difficult diagnosis when occurs under anesthesia. The separation phenomenon of decrease in P_ET_CO_2_ and increase in PaCO_2_ might be a useful and suggestive sign, enabling prompt management and therefore improving the prognosis.

**Electronic supplementary material:**

The online version of this article (doi:10.1186/s12871-017-0360-0) contains supplementary material, which is available to authorized users.

## Background

Acute pulmonary embolism (APE) remains a significant medical problem, relating to more than 300,000 deaths per year in Europe [1]. Early detection and treatment are vital for a better prognosis [2]. Signs and symptoms such as dyspnea, chest pain, hemoptysis and syncope could be clues for APE detection [3, 4]. But they are not available for patients under general anesthesia, which increases the difficulty for early diagnosis and stresses the need for other indications. Moreover, the pathophysiologic course of intraoperative APE is affected by procedures of operation and anesthesia, which might be different from conscious patients with spontaneous respiration. The following report describes a case of APE during hip replacement surgery, that provides us with a meaningful phenomenon—an opposite trend in increased PaCO_2_ and decreased P_ET_CO_2_—as an early sign of potential APE for patients undergoing general anesthesia and mechanical ventilation.

## Case presentation

A 64-year-old, BMI 32.7 kg/m^2^, American Society of Anesesthesiolgists (ASA) physical status III, female was scheduled for right hip replacement because of bone fracture. She received a surgery for bilateral lower extremity varicose veins 8 years ago and recovered well. There is no past medical history of hypertension, diabetes mellitus or cerebrovascular diseases. Patient had no drug or food allergies, never smoked cigarettes or consumed alcohol.

Chest X-ray showed heart shadow enlargement (cardiothoracic ratio: 0.65) and slight pleural effusion left side. Echocardiogram showed bilateral atrial dilatation, atrial fibrillation, slight bicuspid and tricuspid regurgitation, right pulmonary mild hypertension (pulmonary arterial systolic pressure: 51 mmHg) and a decreased systolic function of left ventricular. ECG showed acceleration of ventricular rate, atrial fibrillation and T wave non specific changes. D-dimer was 2.517 μg/ml (normal: 0–0.3 μg/ml). Other laboratory assessments were within normal limits. Despite abnormal findings in pre-procedure tests, no uncomfortable symptoms were reported. She emphasized to be in good condition before the fracture that she could do Dama square dance for 2 h every day after supper. There were no complaints except for the pain due to bone fracture. She denied further examination or treatment and insisted on an operation for the fracture as soon as possible, as it reduced her life quality severely.

Therefore she was scheduled for surgery and transferred to the operating room 3 days after admission. Preoxygen and monitoring were performed, which showed BP 110/80 mmHg, HR 140 bpm and SpO_2_ 98%. Considering her anxiety and tachycardia, 10 mg esmolol and 60 μg dexmedetomidine were given intravenously in 10 min. Soon the heart rate slowed down and stabled at 110 bpm. Arterio radialis puncture was performed for invasive blood pressure monitoring under 1% lidocaine local anesthesia. For anesthesia induction, midazolam (0.03 mg/kg), fentanyl (4 μg/kg), etomidate (0.25 mg/kg) and cisatracurium (0.2 mg/kg) were administered. After intubation, mechanical ventilation started with volume control model (RR:12 breaths per minute, tidal volume:6-8 ml/kg, P_ET_CO_2_:35-45 mmHg). General anesthesia was maintained with sevoflurane (1%), propofol (4-10 mg/kg/h), remifentanil (0.1–0.2 μg/kg/min), cisatracurium (0.1 mg/kg/h) and dexmedetomidine (0.5 μg/kg/h). A central venous catheter was placed in the right internal jugular vein under ultrasonic guidance in case of mass transfusion and vasoactive drugs application intraoperatively. Patient was then placed in left lateral decubitus position and the operation began.

Approximately 1 h later, when the surgery was almost finished and the incision was closing, a sudden pronounced decrease in oxygen saturation (100%–66%) was noticed, along with gradually increased airway pressure(22-33cmH_2_O) and decrease in blood pressure (lowest to 89/60 mmHg). P_ET_CO_2_ decreased from 34 mmHg to 22 mmHg. ECG showed rapid atrial fibrillation (heart rate 140-160 bpm). Auscultation revealed bilateral breath sounds. Central venous pressure (CVP) was assessed immediately with a value of 30cmH_2_O. Instant arterial blood gas analysis showed hypercapnia (Table 1). The opposite trend of PaCO_2_ and P_ET_CO_2_, combined with other signs and medical history, indicated the possibility of APE. Transesophageal echocardiogram (TEE) was arranged at once, which showed enlargement of the right atrial, abnormal echoic area in pulmonary artery (considering thrombus) and pulmonary hypertension (pulmonary arterial systolic pressure: 77 mmHg) (Additional file 1: Movie S1).

**Table 1:** The arterial blood gas analysis of the patient

Parameter	T1	T2
pH	7.161	7.231
pCO_2_(mmHg)	66.9	62.7
pO_2_(mmHg)	40.7	76.4
SO_2_(%)	65.9	92.7
cLac(mmol/L)	0.6	0.6
ctO_2_(mmol/L)	12.6	17.8
p50(mmHg)	32.19	31.75
HCO3std(mmol/L)	18.3	21.8
HCO3^−^(mmol/L)	22.9	25.3
ABE(mmol/L)	−6.8	−3.1
SBE(mmol/L)	−4.6	-1.3

T1: the time point when respiratory and hemodynamic changes occurred. T2: the time point when the patient’s condition became stable. T2 time point was 9 min after the T1 time point



**Additional file 1: Movie S1.** A real-time TEE image revealed a mobile embolus in the PA. PA, pulmonary artery; Ao, aorta. (WMV 1mb)


Multidisciplinary consultation started simultaneously. Mechanical and manual ventilation were controlled alternately. Cedilanid (0.2 mg), phenylephrine (0.03–0.06 mg/kg/min), 5% NaHCO_3_ liquid (100 ml) and methylprednisolone (500 mg) were administered intravenously to maintain hemodynamic stability and improve internal environment. All fluids were limited to reduce the cardiac load. 11 min later, SpO_2_ increased to 95% and BP rose to 120/80 mmHg. A following systematic ultrasound examination revealed deep venous thrombosis, which was not found before the surgery and might be the source of pulmonary embolism. After discussion with surgical and respiratory consultation team and communication with the patient’s family members, low molecular weight heparin calcium(LMWHC) (6000 IU) was given and inferior vena cava (IVC) filter was implanted in DSA operating room. After surgery, the patient was transferred to ICU intubated. Patient recovered her consciousness 3 h later and extubated the next morning. Further treatment including anticoagulation, alleviation of pulmonary hypertension and atrial fibrillation therapy was continued. IVC filter was removed 20 days later. Her condition was improved and all vital signs maintained stable. The patient was discharged from hospital uneventfully one month later.

## Discussion

Here we described a case of intra-operative APE and reported a meaningful sign --“separation phenomenon” during general anesthesia, which means a decrease trend in P_ET_CO_2_ and increase in PaCO_2._ This is different from conscious patients with incomplete obstruction of pulmonary artery, whose physiological dead space increases and total minute ventilation increases correspondingly. In these patients, PaCO_2_ is mostly lower than normal because of the compensatory mechanism. However, this mechanism does not work under general anesthesia and mechanical ventilation. Thus when APE occurred in patients with mechanical ventilation, P_ET_CO_2_ does not actually reflect PaCO_2_ anymore. In fact, the difference of PaCO_2_ and P_ET_CO_2_ could be a representative of dead space, which has been studied as a tool to monitor the effect of thrombolysis [5]. Beside dead space increase, low cardiac output and ventilation-perfusion mismatch also contribute to the hypoxemia and hypercapnia [6]. Though not pathognomonic, the separation phenomenon is still valuable. Just like chest pain and dyspnea, these symptoms are not specific, but in a similar context to our case, could be highly suggestive. P_ET_CO_2_ is a routine monitoring under general anesthesia and arterial blood analysis is a quick bedside test. Thus we suggest strict monitoring of P_ET_CO_2_ and arterial blood analysis especially when P_ET_CO_2_ decreases without other reasons during general anesthesia. When the separation phenomenon occurred, PE should be taken into consideration.

We suspected venous thrombus that led to APE in this patient. First, she had a surgical history of varicosity. Although no thrombus was found before the surgery, it was a risk factor of pulmonary thromboembolism. Second, intraoperative TEE showed abnormal echoic area in pulmonary artery (considering thrombus) and the following systematic ultrasound examination revealed deep venous thrombosis. The most common cause of PE remains deep vein thrombosis [7]. Finally, anti-coagulation therapy was useful to improve the condition.

The initial clinical symptoms in this case could also suggest a pneumothorax. But there was no further evidence. Procedure of central venous of catheterization might be a cause for pneumothorax. But the process was guided by ultrasound without any abnormal sign. Also, instant intraoperative auscultation confirmed bilateral breath sounds.

Since it was a hip surgery, the intra-operative APE might also be caused by cement embolus. The known bone cement implantation syndrome (BCIS) is commonly caused by PE, which is characterized by a number of clinical features such as hypoxia, hypotension, cardiac arrhythmias and increased pulmonary vascular resistance. It usually occurs at one of these stages of surgical procedure including femoral reaming, acetabular or femoral cement implantation, insertion of the prosthesis or joint reduction [8]. However, in this case, the hemodynamic changes and hypoxia presented at none of these stages but at the end of operation, when the wound was closing. There are studies showing that cement embolic events are common and most patients tolerate well [9]. Even if BCIS occurs, there is no specific treatment at present [8]. To confirm the cause of APE might need further test, like computed tomography. It was not conducted in this case, which was a pity, considering the high cost and little effect on the treatment.

Combining all the clues, the most possible reason for the hemodynamic and respiratory changes in this case was pulmonary thromboembolism. The treatment requires rapid and accurate risk assessment. Thrombolytic treatment restores pulmonary perfusion more rapidly than anticoagulation alone, but it also carries a risk of major bleeding, including intracranial hemorrhage. And the increased age and presence of comorbidity have been associated with higher risk of hemorrhage [10]. Surgery is also a relative contraindication to thrombolysis. After discussion with the consultation team and communication with patient’s family members, anticoagulation therapy was started and IVC filter was placed to prevent PE recurrence. The patient finally discharged uneventfully.

The good clinical outcome could be predicted by the lactate value. Plasma lactate concentration is a sensitive marker of tissue hypoxia and persistent tissue hypoxia may cause irreversible cell injury which was associated with the adverse outcome in critically ill patients [11, 12]. Studies showed that plasma lactate was prognostic for short-term PE-related complications and high plasma lactate (≥2 mol/L) was associated with increased in-hospital mortality [13–15]. Lactate concentration in this case remained at a low level which might due to our fast detection and treatment. Thus tissue hypoperfusion was temporary and reversible.

Despite updated guidelines, PE remains an important clinical problem with a high mortality rate. Even with prophylaxis, it could still happen and sometimes appeared as an emergency [16]. A flow diagram specific to intra-operative APE might be a useful aid, especially for this infrequent and high-risk situation. Also, multidisciplinary cooperation is necessary to get the most appropriate treatment strategy.

## Conclusion

Pulmonary embolism is a rare and potentially high-risk perioperative situation, with a difficult diagnosis when occurs under anesthesia. The separation phenomenon of decrease in P_ET_CO_2_ and increase in PaCO_2_ might be a useful and suggestive sign, enabling prompt management and therefore improving the prognosis.

## Abbreviations

APE: Acute pulmonary embolism
CVP: Central venous pressure
DVT: Deep vein thrombosis
IVC: Inferior vena cava
P_ET_CO_2_: End-tidal CO_2_ pressure
TEE: Transesophageal echocardiogram
